# Melioidosis and pulmonary tuberculosis co-infection in a diabetic

**DOI:** 10.4103/1817-1737.62476

**Published:** 2010

**Authors:** Anup Kumar Shetty, Rekha Boloor, Vishnu Sharma, Ganesh Hosahithlu Keshava Bhat

**Affiliations:** *Department of Microbiology, Father Muller Medical College, Kankanady, India*; 1 *Department of Medicine, A. J. Institute of Medical Sciences, Kuntikana, Mangalore, Karnataka, India*

**Keywords:** *Burkholderia pseudomallei*, melioidosis, tuberculosis

## Abstract

*Burkholderia pseudomallei* is the causative agent of melioidosis. It is endemic in South East Asian countries and North Australia. Sporadic cases of melioidosis have been reported from several parts of South India. Melioidosis may manifest as chronic pneumonia mimicking tuberculosis and generally be seen as a single entity. We report the first case of melioidosis and pulmonary tuberculosis co-infection in a diabetic patient. The causative agents were identified using standard methods and the patient recovered after completion of the recommended antibiotic therapy. Melioidosis is an emerging infectious disease in India. Though melioidosis and tuberculosis present with similar clinical picture, co-infections are rare. Hence, increased awareness among clinicians and microbiologists can help in diagnosing the disease even when there is no clinical suspicion.

Melioidosis is caused by *Burkholderia pseudomallei (B. pseudomallei)* which manifests as an asymptomatic infection, localized skin ulcer or abscess, or chronic pneumonia mimicking tuberculosis and fulminant septic shock with abscess in multiple internal organs.[1] Pneumonia due to *B. pseudomallei* itself is a rare condition and co-infection with tuberculosis is extremely rare. *B. pseudomallei* is widely distributed in soil and stagnant waters of endemic areas like South East Asia and northern Australia.[1–3] Only sporadic reports of melioidosis from various parts of South India and coastal India are seen, and there is a lack of awareness about this disease.[4] Tuberculosis is endemic in developing countries like India. Melioidosis is now increasingly recognized and is an emerging infectious disease in India.[4,5]

## Case Report

We report a case of melioidosis and pulmonary tuberculosis co-infection in a 40-year-old diabetic male. The patient presented with chronic cough since two months and intermittent low grade fever since a month. Cough was initially dry and later productive, with no hemoptysis. The patient was initially managed as an outpatient in his village with symptomatic treatment for cough and fever, following which he had temporary relief. A diabetic for 13 years, he was on irregular treatment. He was a non-smoker and non-alcoholic. He was an agriculturist by profession, cultivating rice paddy. He had no past history of tuberculosis and bronchial asthma.

On examination, his temperature was 39.4°C. Dyspnoea, clubbing or lymph node enlargement were not present. Fine crepitations were heard on left upper lobe area of lung. Laboratory investigations showed a total leukocyte count, 8,700/mm^3^ (normal range, 4,000-10,500) with 68% neutrophils, 1% monocytes, 26% lymphocytes and eosinophils 5%, Erythrocyte sedimentation rate 20 mm/1^st^ hr (normal range, 2-14 mm/ 1^st^ hr), random blood sugar 420mg/dl (normal range, 80-160 mg/dl). Hemoglobin level, liver function test, thyroid function test, renal function tests were within normal limits. Chest X-ray showed cavitory lesion in left apical lobe and no consolidation. Ultrasonography of abdomen was normal.

Three sputum samples collected on three consecutive days were cultured and *Burkholderia pseudomallei* (*B. pseudomallei*) was isolated in all the cultures. Identification was done by standard techniques using Gram's staining and biochemical reactions.[6] Antibiotic susceptibility was performed and interpreted as per Clinical Laboratory Standards Institute guidelines.[7] Acid fast bacilli (AFB) staining by Ziehl-Neelsen's method was negative for all three sputum samples.[6] *Mycobacterium tuberculosis* was not isolated in any sputum culture.[6] A bronchoscopy was done three days later, and *B. pseudomallei* was isolated from culture of bonchoscopic aspirate. The aspirate was also positive for AFB and hence the specimen was inoculated in Lowenstein-Jensen media for Mycobacterial culture. *Mycobacterium tuberculosis* was isolated in culture after five weeks of incubation.[6] Blood culture was negative for both *B. pseudomallei* and *Mycobacterium tuberculosis*. As *B. pseudomallei* was repeatedly isolated in different specimens - three sputum and one bronchoscopic aspirate, melidiosis was proven beyond doubt. Tuberculosis was established after *Mycobacterium tuberculosis* was isolated from bronchoscopic aspirate culture.

For melioidosis, the patient was started on an intensive therapy with ceftazidime (two grams every six hours) and co-trimoxazole (sulphamethoxazole/trimethoprim, 800/160mg every 12 hours) for two weeks, followed by maintenance therapy with doxycycline (100mg every 12 hours) and co-trimoxazole (sulphamethoxazole/trimethoprim, 800/160mg every 12 hours) for three months.[1] He was also administered standard multidrug therapy for tuberculosis for a period of six months consisting of Rifampicin (600mg/day), Isoniazid (300mg/day), Pyrazinamide (1.5 gram/day), ethambutol (800mg/day) for a period of two months followed by Rifampicin (600mg/day) and Isoniazid (300mg/day) for a period of four months.[1,6] His liver function was monitored weekly for two months and later on for every 15 days. It was within normal limits throughout the course of treatment. The patient became symptom free within 10 days of commencement of therapy and recovered completely at the end of therapy.

## Discussion

Melioidosis is a disease of humans and animals. *B. pseudomallei* is a Gram negative, aerobic, non-fermenting bacillus, previously called as *Pseudomonas pseudomallei.*[6] Melioidosis is endemic in South East Asia and northern Australia, with largest number of reports from Thailand.[1–4] Sporadic cases of melioidosis have been reported from various parts of Southern India and is now an emerging infectious disease.[1–5,8] *B. pseudomallei* is found more commonly in irrigated sites such as rice paddies and surface waters of endemic areas, facing heavy rainfall.[1,5] Majority of the reported cases of melioidosis from endemic areas and from sporadic cases of South India occurred in monsoonal wet seasons,[3,4,8] which was observed in this case also.

*B. pseudomallei* can be grown easily by standard laboratory methods. When isolated it is often mistaken for *Pseudomonas species* and either treated as such or ignored as contaminants.[5] Melioidosis is under reported, probably because of lack of awareness among the clinicians and microbiologists.[4]

Modes of transmission include inhalation, direct contact with infected rodents or food, soil, water, excreta, inoculation from contaminated soil through abrasions or lesions.[2,9] Major risk factors for melioidosis are diabetes mellitus, excess alcoholism and renal disease. Other risk factors include chronic lung disease, thalassemia, malignancies, steroid therapy, iron overload and tuberculosis.[1,10] This patient had a risk factor of diabetes mellitus, but he did not have any past history of tuberculosis and hence both infections had presented for first time. Melioidosis may manifest as asymptomatic infection, localized skin ulcer or abscess, chronic pneumonia mimicking tuberculosis and fulminant septic shock with multiple abscess in internal organs.[1] In Indian reports melioidosis presented either as pneumonia, cellulitis, osteomyelitis, abscess in internal organs or septicemia. Pneumonia was the commonest presentation.[2,4,5,8] None of them reported co-existence of active tuberculosis and melioidosis. Hence, to the best of our knowledge, this is the first case to be reported from India.

In melioidosis, patients may have features mimicking tuberculosis, with fever, weight loss, productive cough, upper lobe infiltrates with or without cavitations on chest radiograph.[1] Hence an initial diagnosis of tuberculosis is usually done. This patient was also initially diagnosed with tuberculosis based on clinical and radiological findings. Since *B. pseudomallei* was isolated in culture he was started on therapy for melioidosis. A broncoscopic aspirate done later revealed co-existence of both *B. pseudomallei* and *Mycobacterium tuberculosis*. Since the patient was not diagnosed or treated for tuberculosis earlier, it is the first infection. *B. pseudomallei* as such can cause a cavity or can colonize in previously existing tubercular cavity.[1] Our patient was an agriculturist cultivating rice paddy and a diabetic living in a nonendemic area facing heavy monsoon. *B. pseudomallei* may have caused the cavity itself or colonized tubercular cavity, causing infection later. As the patient was not investigated previously it is difficult to say which infection appeared first. But patient presented with symptoms for first time and both organisms were identified simultaneously for first time. As both diseases present with similar clinical picture, co-existing tuberculosis may be missed once diagnosed as melioidosis. Without a bronchoscopic aspirate we would have missed co-existing tuberculosis infection in this patient. Hence it may be appropriate to do a bronchoscopy to rule out tuberculosis co-infection in melioidosis, if sputum is negative for AFB.

## Conclusion

Melioidosis is an emerging infectious disease in India. Patients with risk factors for melioidosis and those from non-endemic areas facing heavy monsoons, presenting with clinical features of tuberculosis, must be thoroughly investigated to rule out melioidosis and co-infections with tuberculosis. An increased awareness among the microbiologists can identify the causative agents, even when there is no clinical suspicion. Successful cure from the disease can be achieved by following the recommended antibiotic regimen.
